# Evaluation of Absolute Neutrophil Count in the Perioperative Setting of Sarcoma Resection

**DOI:** 10.1155/2024/4873984

**Published:** 2024-01-24

**Authors:** Gayathri Vijayakumar, Emma M. Steffer, Neil P. Buac, Matthew W. Colman, Steven Gitelis, Alan T. Blank

**Affiliations:** Department of Orthopedic Surgery, Division of Orthopedic Oncology, Rush University Medical Center, Chicago, Illinois, USA

## Abstract

**Introduction:**

Limb salvage surgery (LSS) is the preferred surgical treatment for bone sarcomas. Preoperatively, many patients receive chemotherapy and may develop neutropenia. No study has evaluated the effect of a low preoperative absolute neutrophil count (ANC) on postoperative outcomes following LSS.

**Methods:**

This was a retrospective review of 114 patients who underwent LSS for bone sarcoma from 2010 to 2020. Preoperative lab values were analyzed by logistic regression to identify the risk of developing surgical complications within 30 days, surgical site infection (SSI), and reoperation.

**Results:**

Three (2.6%) patients experienced a surgical complication within 30 days. Twelve (10.53%) patients experienced postoperative SSI. Twenty-nine (25.4%) required reoperation. Preoperative ANC was not a significant predictor of surgical complications within 30 days, SSI, or reoperation. The only independent predictor of worse overall survival was the presence of a pathologic fracture at the time of surgery.

**Conclusion:**

This is the first study to evaluate preoperative ANC on postoperative outcomes following LSS. We report no significant differences in surgical complications within 30 days, SSI, or reoperation with low preoperative ANC. Future studies with larger cohorts of neutropenic patients are needed to evaluate these outcomes, as our cohort had very few neutropenic patients due to selection bias.

## 1. Introduction

Bone sarcomas are a collection of rare cancers derived from mesenchymal cells with an incidence of approximately 3,900 new cases a year [1]. The most common primary bone sarcomas are osteosarcoma, chondrosarcoma, and Ewing sarcoma; however, leiomyosarcoma (LMS) and undifferentiated pleomorphic sarcoma (UPS) occasionally arise as primary tumors in the bone [2–4]. Of these, osteosarcoma is the most common primary malignancy of bones. Treatment of bone sarcomas includes neoadjuvant chemotherapy followed by surgical resection and adjuvant chemotherapy. Limb salvage surgery (LSS) has become the preferred method of surgical resection over amputation and is associated with better functional outcomes [5, 6].

When proceeding with LSS after chemotherapy, it is important to consider the surgical risks to the patient. Certain chemotherapy regimens used in the treatment of sarcomas can cause myelosuppression with a reduction in absolute neutrophil count (ANC) [7]. Neutropenia is defined as ANC below 1500 cells per microliter (*μ*L) and is associated with increased susceptibility to infections, diminished inflammatory response, and higher mortality [8, 9]. Neutropenic patients undergoing abdominal surgery have demonstrated higher rates of postoperative complications, surgical site infections (SSI), and morbidity [10–12]. Zarain-Obrador et al. [12] reported preoperative neutropenia as an independent predictor of SSI in 1,727 colorectal surgeries. In a study of 237 neutropenic patients receiving abdominal surgery, Jolissaint et al. [10] reported that lower preoperative ANC was significantly associated with 30-day mortality. In the orthopedic oncology literature, one study evaluated preoperative WBC on tibial allograft reconstruction infection rates and reported a statistically significant increase in infection with decreased total white blood cell count (WBC) [13].

Surgical recommendations for neutropenic oncology patients suggest postponing surgery until the ANC has normalized, especially if the ANC is below 1000 [14, 15]. In addition, there are no studies on ANC's effects on the surgical management of sarcoma of the bone specifically. However, waiting for ANC to recover in cases of neutropenia involves further time off from chemotherapy which risks growth or progression of the malignancy. The goal of this paper is to evaluate postoperative complications related to preoperative ANC count to further guide treatment courses for sarcoma of the bone. We hypothesize that lower preoperative ANC leads to increased postoperative complications including surgical complications within thirty days, surgical site infections, and reoperations.

## 2. Materials and Methods

Following Institutional Review Board' approval, we performed a retrospective review of all patients who underwent limb salvage surgery for osteosarcoma, Ewing's sarcoma, chondrosarcoma, LMS bone, and UPS bone from 2010 to 2020. Patients with no preoperative ANC count, no record of limb salvage surgery, and incomplete or missing information regarding postoperative outcomes were excluded. The records of 114 patients met these criteria and were included.

Demographic and clinicopathological information including sex, age, body mass index, smoking history, primary tumor type, presence of the pathologic fracture at diagnosis, and treatment with chemotherapy and radiotherapy were collected from the medical record. Preoperative lab values including absolute neutrophil count (ANC), absolute lymphocyte count (ALC), albumin, and creatinine closest to the date of surgery, operative characteristics, and postoperative outcomes were also collected from the medical record. Overall survival (OS) was defined as the time from the date of surgery to the date of the last follow-up or death from any cause.

### 2.1. Statistical Analysis

Continuous variables were described as medians with an interquartile range (IQR) and compared using the Mann–Whitney *U* test. Categorical variables were described as totals and frequencies and compared using the *χ*^2^ test or Fisher's exact test as appropriate. Bivariate logistic regression analyses were performed to assess the effect of preoperative lab values on postoperative outcomes: surgical complications within 30 days, surgical site infection, and reoperation. They were expressed as odds ratios (OR) and 95% confidence intervals (CI). The final model(s) were checked for goodness of fit with the Hosmer and Lemeshow tests to ensure they were well specified and fit the data [16]. Associations between demographics, preoperative labs, operative characteristics, and postoperative outcomes with OS were analyzed with Kaplan–Meier curves and log-rank testing. Cox proportional hazards regression was used to determine prognostic factors in univariable and multivariable analyses, expressed as hazard ratios (HR) and 95% CI. Factors that were statistically significant in the univariable analysis were included in the multivariable model. Statistical significance was set to *p* < 0.05, and all analyses were performed on SPSS version 26.0 (IBM, Armonk, NY, USA).

## 3. Results

### 3.1. Overall Demographics

One hundred fourteen patients who underwent LSS for osteosarcoma, Ewing's sarcoma, chondrosarcoma, LMS bone, and UPS bone from 2010 to 2020 were included in the study. Demographic and clinical characteristics are detailed in Table 1. The median age at presentation was 36 years (IQR 18–55.25), and 51.8% of patients were male. Twenty-eight (24.6%) patients had a smoking history. The most common tumor type was osteosarcoma (43.9%) followed by chondrosarcoma (36%), Ewing's sarcoma (11.3%), UPS bone (4.4%), and LMS bone (4.4%). Twenty-three patients (20.2%) received perioperative radiotherapy and 68 patients (59.6%) received perioperative chemotherapy. The median preoperative ANC count was 4.10 thousand cells per *μ*L (IQR 2.58–6.00). The median preoperative ALC was 1.62 (1.18–2.09). Only four patients had preoperative ANC below 1000. Twenty patients had a preoperative ALC below 1000. The most common limb salvage procedure was endoprosthetic reconstruction (43.9%) followed by resection or intralesional excision (29.8%). Operative characteristics and postoperative outcomes are illustrated in Table 2. The median operative time was 175.50 minutes (IQR 123.25–270.75). The median follow-up was 35.5 months (IQR 21.75–58.25). Two patients (1.7%) experienced severe blood loss intraoperatively. Thirty-one (27.2%) patients experienced thirty-two postoperative complications with deep surgical site infection (9.6%) and graft nonunion (7.9%) as the two most common complications.

## 4. Complications

### 4.1. Preoperative ANC Count above 1000 vs below 1000

Table 3 illustrates postoperative complications based on preoperative ANC above or below 1000 cells per *μ*L. The median number of days from preoperative labs to LSS was eight days. Only one case of graft fracture occurred in a patient with preoperative ANC below 1000 (*p*=0.035). The remaining complications included 12 patients with surgical site infection (SSI) (*p*=1.00), nine patients with graft nonunion (*p*=1.00), six patients with prosthesis failure (*p*=1.00), three patients with a surgical complication within 30 days (*p*=1.00), and one patient who returned to the operating room (OR) in 30 days from a surgical complication (*p*=1.00) occurred in patients with preoperative ANC above 1000. Only two additional patients had a preoperative ANC below 1500, and there were no significant differences in complications between patients with ANC below 1500 compared to those above 1500. No significant difference in preoperative ANC was found between patients of different races (*p*=0.850).

### 4.2. Preoperative ALC Count above 1000 vs below 1000

Table 4 illustrates postoperative complications based on preoperative ALC above or below 1000 cells per *μ*L. Only one case of graft fracture occurred in a patient with a preoperative ALC below 1000 (*p*=0.175). The remaining complications in patients with ALC below 1000 include four patients with SSI (*p*=0.219), two patients with graft nonunion (*p*=0.657), and two patients with surgical complications within 30 days (*p*=0.079). No significant difference in preoperative ALC was found between patients of different races (*p*=0.385).

### 4.3. Surgical Complication within 30 Days

Three patients (2.6%) experienced surgical complications within 30 days which were three deep SSI. Hypertension was significantly associated with surgical complications within 30 days (*p*=0.011). Perioperative chemotherapy was not significantly associated with surgical complications within 30 days (*p*=1.00). The median ANC for these three patients was 2.32 thousand cells per *μ*L (IQR 2.19–3.34) compared to 4.11 thousand cells per *μ*L (IQR 2.71–6.02) in the other patients, and this difference was insignificant (*p*=0.234). The median ALC for these three patients was 0.91 thousand cells per *μ*L (IQR 0.90–1.81) compared to 1.62 thousand cells per *μ*L (IQR 1.22–207) in the other patients and this difference was insignificant (*p*=0.651). On logistic regression analysis, we could not find any significant predictors of surgical complications within thirty days based on preoperative lab values including prechemo or baseline ANC in patients who received chemotherapy, preoperative ANC, and preoperative ALC. We report that the odds of surgical complication within 30 days decreased by roughly 80% with every 1000 unit increase in ANC (OR = 0.210; 95% CI 0.028–1.527; *p*=0.119); however, this was statistically insignificant.

### 4.4. Surgical Site Infection

Twelve (10.5%) patients experienced surgical site infections following LSS. The median preoperative ANC in patients with postoperative SSI was 2.74 thousand cells per *μ*L (IQR 2.29–5.37) compared to 4.26 thousand cells per *μ*L (IQR 2.80–5.98) in patients without postoperative SSI, and this difference was insignificant (*p*=0.433). The median preoperative ALC in patients with postoperative SSI was 1.57 thousand cells per *μ*L (IQR 0.91–1.95) compared to 1.62 thousand cells per *μ*L (IQR 1.22–2.07) in patients without postoperative SSI, and this difference was insignificant (*p*=0.605). Perioperative chemotherapy was not significantly associated with SSI (*p*=0.355). On logistic regression analysis, we were unable to identify statistically significant predictors of SSI based on preoperative lab values including prechemo or baseline ANC in patients who received chemotherapy, preoperative ANC, and preoperative ALC.

### 4.5. Reoperation

Twenty-nine (25.4%) patients received reoperation for graft or prosthetic complications and local recurrence. Preoperative ANC in patients with reoperation was not significantly different (*p*=0.178) compared to those without reoperation. Preoperative ALC in patients with reoperation was not significantly different (*p*=0.960) compared to those without reoperation. Allograft reconstruction was significantly associated with reoperation (*p*=0.003). Perioperative chemotherapy was not significantly associated with reoperation (*p*=0.236). On logistic regression analysis, we found no significant predictors of reoperation based on preoperative lab values including prechemo or baseline ANC in patients who received chemotherapy, preoperative ANC, and preoperative ALC.

### 4.6. Overall Survival

The median OS was 44 months (IQR 26–65). OS for the entire cohort was 93.8% at 1 year, 81.6% at three years, and 74.4% at five years. On univariate analysis, worse OS was significantly associated with metastasis at the time of surgery (*p*=0.005), pathologic fracture (*p*=0.025), low vs intermediate/high tumor grade (*p*=0.019), perioperative radiotherapy (0.011), increasing operative duration (0.024), and longer postoperative length of stay (*p*=0.024). On multivariate analysis, only pathologic fracture (HR 2.990 (95% CI 1.099–8.135); *p*=0.032) remained predictive of worse OS. Tumor grade (HR 2.183 (95% CI 0.450–10.593); *p*=0.333), metastasis at time of surgery (HR 2.845 (95% CI 0.753–8.199); *p*=0.135), perioperative radiotherapy (HR 2.257 (95% CI 0.928–5.845); *p*=0.073), operative time (HR 1.002 (95% CI 0.999–1.006); *p*=0.179), and postoperative length of stay (HR 0.954 (95% CI 0.804–1.131); *p*=0.587) were not predictive of worse OS on multivariate analysis (Table 5).

## 5. Discussion

The management of bone sarcomas is often a multidisciplinary effort involving the entire musculoskeletal oncology team. Neoadjuvant chemotherapy is an important part of the presurgical treatment; however, certain chemotherapy regimens can cause myelosuppression and reduce white blood cell counts, specifically ANC. For surgical management of bone sarcoma, orthopedic oncologists must consider potential risks with surgery in patients with low preoperative ANC. Lower preoperative ANC has been associated with higher rates of postoperative complications, infections, and morbidity in patients undergoing abdominal surgery [10–12]. As there are no studies reporting the effects of preoperative ANC on LSS of bone sarcoma, we sought to investigate the effects of preoperative ANC on surgical complications within 30 days, SSI, and reoperation in this cohort.

In conducting this study, we hoped to evaluate postoperative outcomes following LSS based on the patient's preoperative ANC as several orthopedic oncology groups strongly advocate for a preoperative ANC threshold prior to surgery. In addition, this is the first study evaluating the effect of preoperative ANC levels on postoperative outcomes following LSS. Although we hypothesized lower preoperative ANC would predict surgical complications within 30 days, SSI, and reoperation, it was a statistically insignificant predictor of these outcomes in our cohort likely due to selection bias. In addition, the prechemo or baseline ANC in patients who received chemotherapy was not a statistically significant predictor of these postoperative outcomes. However, the median of ANC and ALC in patients with surgical complications within 30 days and SSI was lesser compared to the median ANC in patients without surgical complications within 30 days and SSI. Interestingly, we found no significant difference in 30-day complications or postoperative infections in patients who were receiving chemotherapy compared to those who were not. In our cohort of 114 patients, only four patients received surgery with an ANC below 1000. Three of these patients had osteosarcoma and one patient had Ewing's sarcoma. All patients were receiving neoadjuvant chemotherapy, and their preoperative lab values were collected one to seven days before surgery. Only one patient experienced a postoperative complication of graft fracture 16 months following allograft reconstruction of their tibia. In our institution, there is a bias to not operate on patients with an ANC below 1000 which is reflected in our cohort. The median preoperative ANC of our cohort was 4.10 (IQR 2.58–6.00) which is within the normal ANC levels. Future studies with a greater number of neutropenic patients undergoing LSS are necessary to evaluate the effects of low preoperative ANC on postoperative outcomes. Furthermore, future studies should evaluate genetic markers as potential prognostic markers in patients with sarcoma following LSS. Previous studies have demonstrated the expression of programmed death-ligand 1 (PDL1), tumor protein 53 (TP53), and MYC protooncogene (MYC) in sarcomas and as potential targets for immunotherapy [17–19]. Given the altered expression of these markers in sarcomas, future studies should evaluate the prognostic ability of these markers.

Surgical site infections are a common complication following LSS in patients, given the complexity of the surgical procedure and the immunocompromised condition of a majority of these patients [20, 21]. In our study, the most common postoperative complication was SSI with three (2.63%) cases of SSI in the first 30 days. Overall, twelve patients (10.5%) experienced SSI with four deep SSIs, seven prosthetic joint infections, and one superficial SSI. This is comparable to the 13.3% infection rate reported by Shehadeh et al. [22] in 232 patients following endoprosthetic reconstruction of sarcoma at a mean 10-year follow-up. The four cases of deep SSI included an abscess formation after en bloc pelvic resection, infected hematoma after en bloc scapular resection, seroma formation after pelvic allograft reconstruction, and sinus tract following femoral en bloc resection. The first three of these deep SSI's occurred within 30 days after surgery. The prosthetic joint infections included three femoral endoprostheses, two tibial endoprostheses, one infected tibial allograft, and one infected pelvic allograft. The one case of superficial SSI occurred after a femoral endoprosthetic reconstruction. The majority of our complications occurred in patients following lower extremity salvage surgery which is supported by the literature [20, 23, 24]. This difference is likely the result of anatomic differences in the lower extremity that contribute to the difficulty of surgical resection in this location.

Following the index limb salvage procedure, complications led to reoperations in 29 patients. Common causes for reoperation following LSS include infection, graft nonunion, graft fracture, prosthesis failure, and local recurrence [25, 26]. The second most common complication in our cohort was graft nonunion. Graft nonunion occurred in three tibial allografts, three femoral allografts, and three humeral allografts at a median of 12.5 months. As mentioned above, graft infections occurred in one pelvic allograft and one tibial allograft while prosthetic infections occurred in three femoral endoprostheses and two tibial endoprostheses. A graft fracture occurred in one tibial allograft. The complications of utilizing allografts for limb reconstruction including infection, nonunion, and fracture have been reported by several studies in the literature for femoral, tibial, pelvic, and humeral allografts [27–31]. Prosthesis failure occurred in three tibial endoprostheses and three femoral endoprostheses at a median of 25.5 months. In a retrospective review of 232 patients receiving endoprosthetic reconstruction for malignant bone tumors, Shehadeh et al. [22] reported lower prosthesis survival in lower extremity prostheses compared to upper extremity prostheses which is consistent with our findings. Local recurrence of the sarcoma after the index limb salvage procedure necessitated reoperation in six (5.2%) patients which is comparable to other reports in the literature [22, 26, 32]. Additional surgical management for infections following the limb salvage procedure in our cohort included irrigation and debridement (I&D) for the sinus tract after femoral en bloc resection, I&D for the seroma after pelvic allograft reconstruction, and interventional radiology-guided draining for the abscess that formed after pelvic resection. Furthermore, a closed knee manipulation was performed on a patient who developed arthrofibrosis following femoral endoprosthetic reconstruction.

Overall survival of bone sarcomas following LSS has improved with advancing surgical and medical oncology treatments. In a cohort of 100 bone and soft tissue upper and lower extremity sarcomas, Quill et al. [26] reported 86% survival at 45 months following LSS. However, other studies have reported varying OS rates from 30% to 80% following limb salvage surgery for sarcoma [33–37]. In our cohort, median OS was 44 months (IQR 26–65) with 74.4% OS at five years follow-up. The only independent predictor of worse OS following LSS was pathologic fracture at the time of surgery which is consistent with the literature [38, 39].

This study has several limitations. This was a retrospective study and is subject to the biases inherent in retrospective analysis. In addition, patients who met inclusion criteria had to be excluded because of the lack of preoperative ANC prior to LSS, which may have introduced selection bias. Although blood counts at or closest to the date of surgery were utilized, there was some variability in the timing and patients may have had different values on different days. Future prospective studies should involve a more uniform cohort which would eliminate much of this variability.

## 6. Conclusion

Although our study found no statistical significance associating ANC with perioperative complications, our group encourages discussion with a multidisciplinary care team to weigh the risks and benefits of performing surgery in the setting of ANC under 1000 versus prolonged time off chemotherapy in order to allow ANC recovery. We do believe ANC is an important variable in the risk profile of developing SSI and warrants further investigation. We hope this study can provide a motivational background for future investigations into a multicenter prospective project investigating this question.

## Figures and Tables

**Table 1:** Demographics.

	*N* (%)
Age at operation (years), median (IQR)	36 (18–55.25)
BMI at operation, median (IQR)	25.61 (22.07–29.95)
Sex	
Male	59 (51.8)
Female	55 (48.2)
Smoking history	28 (24.6)
Primary tumor type	
Ewing's sarcoma	13 (11.3)
Undifferentiated pleomorphic sarcoma, bone	5 (4.4)
Leiomyosarcoma, bone	5 (4.4)
Osteosarcoma	50 (43.9)
Chondrosarcoma	41 (36.0)
Pathologic fracture	17 (15.0)
Metastases	10 (8.8)
Perioperative chemotherapy	68 (59.6)
Perioperative radiation	23 (20.2)
Preoperative ANC (thousand per *μ*L), median (IQR)	4.10 (2.58–6.00)
Preoperative ALC (thousand per *μ*L), median (IQR)	1.62 (1.18–2.09)
Salvage procedure	
Adult endoprosthesis	48 (42.1)
Expandable endoprosthesis	2 (1.8)
Allograft reconstruction	25 (21.9)
Autograft reconstruction	3 (2.6)
Rotationplasty	2 (1.8)
Resection or intralesional excision	34 (29.8)

IQR, interquartile range; BMI, body mass index; ANC, absolute neutrophil count; *μ*L, microliter; ALC, absolute lymphocyte count.

**Table 2:** Operative characteristics and postoperative information.

	*N* (%)
Operative time (minutes), median (IQR)	175.50 (123.25–270.25)
Estimated blood loss (mL), median (IQR)	300 (150–500)
Intraoperative complications	2 (1.8)
Length of stay (days), median (IQR)	4 (2–6)
Postoperative complications	31 (27.2)
Superficial surgical site infection	1 (0.9)
Deep surgical site infection	11 (9.6)
Graft fracture	1 (0.9)
Graft nonunion	9 (7.9)
Prosthesis failure	6 (5.3)
Peroneal nerve injury	2 (1.8)^*∗*^
Arthrofibrosis	1 (0.9)
Urologic complication	1 (0.9)
Reoperation	29 (25.4)
Surgical complication within 30 days	3 (2.6)
Return to OR in 30 days	1 (0.9)

IQR, interquartile range; mL, milliliters; OR, operating room; ^*∗*^one case of peroneal nerve injury in patient with deep SSI.

**Table 3:** Postoperative outcomes based on preoperative ANC levels.

	ANC below 1000	ANC above 1000	*p* value
Total	4	110	
Surgical site infection	0	12	1.00
Graft fracture	1	0	**0.035**
Graft nonunion	0	9	1.00
Prosthesis failure	0	6	1.00
Surgical complication within 30 days	0	3	1.00
Return to OR in 30 days	0	1	1.00

ANC, absolute neutrophil count; OR, operating room; statistically significant *p* values are bolded.

**Table 4:** Postoperative outcomes based on preoperative ALC levels.

	ALC below 1000	ALC above 1000	*p* value
Total	20	94	
Surgical site infection	4	8	0.219
Graft fracture	1	0	0.175
Graft nonunion	2	7	0.657
Prosthesis failure	0	6	0.588
Surgical complication within 30 days	2	1	0.079
Return to OR in 30 days	0	1	1.00

ALC, absolute lymphocyte count; OR, operating room.

**Table 5:** Univariate and multivariate analyses for overall survival.

	Univariate analysis	*p* value	Multivariate analysis	*p* value
Tumor grade (low vs intermediate/high)	HR 4.798 (95% CI 1.128–20.145)	**0.019**	HR 2.183 (95% CI 0.450–10.593)	0.333
Metastasis at time of surgery	HR 3.397 (95% CI 1.366–8.449)	**0.005**	HR 2.845 (95% CI 0.753–8.199)	0.135
Pathologic fracture	HR 2.700 (95% CI 1.130–6.452)	**0.025**	HR 2.990 (95% CI 1.099–8.135)	**0.032**
Perioperative chemotherapy	HR 1.619 (95% CI 0.711–3.684)	0.251		
Perioperative radiotherapy	HR 2.692 (95% CI 1.259–5.756)	**0.011**	HR 2.257 (95% CI 0.928–5.845)	0.073
Preoperative ANC (below vs above 1000)	HR 0.689 (95% CI 0.093–5.115)	0.714		
Preoperative ALC (below vs above 1000)	HR 0.753 (95% CI 0.284–2.001)	0.570		
Operative time	HR 1.003 (95% CI 1.00–1.005)	**0.024**	HR 1.002 (95% CI 0.999–1.006)	0.179
Length of stay	HR 1.069 (95% CI 1.013–1.128)	**0.024**	HR 0.954 (95% CI 0.804–1.131)	0.587
Postoperative complications	HR 0.758 (95% CI 0.321–1.794)	0.529		
Surgical complication within 30 days	HR 2.449 (95% CI 0.575–10.426)	0.225		
Return to OR in 30 days	HR 0.049 (95% CI 0.000–2037084.398)	0.736		
Reoperation	HR 0.379 (95% CI 0.131–1.094)	0.073		

ANC, absolute neutrophil count; ALC, absolute lymphocyte count; OR, operating room; statistically significant *p* values are bolded.

## Data Availability

The data that support the findings of this study are available from the corresponding author upon reasonable request.
